# Tackling the Burden of Electronic Health Record Use Among Physicians in a Mental Health Setting: Physician Engagement Strategy

**DOI:** 10.2196/32800

**Published:** 2022-03-08

**Authors:** Tania Tajirian, Damian Jankowicz, Brian Lo, Lydia Sequeira, Gillian Strudwick, Khaled Almilaji, Vicky Stergiopoulos

**Affiliations:** 1 Information Management Group Centre for Addiction and Mental Health Toronto, ON Canada; 2 Department of Family and Community Medicine Faculty of Medicine University of Toronto Toronto, ON Canada; 3 Institute of Health Policy, Management and Evaluation University of Toronto Toronto, ON Canada; 4 Centre for Complex Interventions Centre for Addiction and Mental Health Toronto, ON Canada; 5 Physician-in-Chief Office Centre for Addiction and Mental Health Toronto, ON Canada; 6 Department of Psychiatry Faculty of Medicine University of Toronto Toronto, ON Canada

**Keywords:** burnout, organizational strategy, electronic health record use, clinical informatics, medical informatics

## Abstract

The burden associated with using the electronic health record system continues to be a critical issue for physicians and is potentially contributing to physician burnout. At a large academic mental health hospital in Canada, we recently implemented a Physician Engagement Strategy focused on reducing the burden of electronic health record use through close collaboration with clinical leadership, information technology leadership, and physicians. Built on extensive stakeholder consultation, this strategy highlights initiatives that we have implemented (or will be implementing in the near future) under four components: engage, inspire, change, and measure. In this viewpoint paper, we share our process of developing and implementing the Physician Engagement Strategy and discuss the lessons learned and implications of this work.

## Introduction

### Background

With growing levels of clinician burnout both before and during the COVID-19 pandemic, the burden associated with the use of electronic health record (EHR) systems has emerged as a paramount challenge [1]. In particular, with an increase in reporting and clinical research needs required by consumers of health data (ie, administrators and researchers) during the spread of the pandemic, there have been additional burden for data generators (ie, providers who document care in the EHR) [2]. This has resulted in clinicians spending more time using the EHR system to complete documentation than actual patient care. The recent call to action by Shanafelt [3] highlighted the importance of thinking about how digital technologies are being introduced as another component into the clinical environment. In particular, there is a greater demand to think about better designed solutions that fit the needs of clinicians [3]. In addition to reports by physicians on the impact of EHR burden on burnout [4,5], this has resulted in numerous recommendations from organizations such as the Ontario Medical Association [6] on reconsidering the impact and use of technology in clinical practice. Therefore, it is likely that EHR-related burden, in addition to the efforts and challenges involved in managing the pandemic, have collectively led clinician burnout to an all-time high [7,8].

As EHR systems are increasingly enhanced with advanced features to improve patient care, such as clinical decision support and predictive analytics, the impact of these capabilities on clinicians’ ability to effectively and meaningfully deliver care must not be forgotten. As highlighted in many commentaries on this topic and most recently, in a chapter on EHR burden by the National Academies of Sciences, Engineering, and Medicine [9], the use of the EHR must not detract from the core aspects of medicine, such as the therapeutic relationship, nor cause unnecessary frustration, complexity, and burden on clinicians [10]. In this context, many initiatives in this field have recently emerged. For example, the *Clickbusters* initiative, developed by the Vanderbilt Clinical Informatics Centre [11], aims to reduce the number of unnecessary alerts delivered to end users, thereby reducing EHR burden and burnout.

Of note, most efforts to date that address EHR-related burden for clinicians have focused on the US context, with scant evidence emerging from other countries. Given that each country has varying practice and clinical requirements, the factors and bottlenecks that influence EHR burden in various settings are likely different. For example, in a recent survey on EHR burnout conducted as part of the 25×5 Symposium [12] in the United States, documentation requirements for reimbursement were cited as a main factor leading to EHR burden for physicians. These challenges are specific to the US context and do not apply to many other countries, including Canada, where health care is publicly funded. However, even without such reimbursement-driven documentation requirements in Canada, we face substantial EHR-related burnout rates [13] comparable with those in other countries. In addition, it is expected that the challenges associated with EHR burden differ across disciplines. For example, given that mental health relies heavily on narrative documentation, the pain points in using the EHR would likely differ from those experienced by specialties that rely on the use of forms or structured templates. Without sufficient discussion and evidence in the literature on how mental health care organizations in Canada are managing these challenges, there is a lack of guidance for these organizations to build relevant and effective initiatives in their own settings. Moreover, it is unclear how existing best practices for system development and implementation (eg, super users) should be best leveraged to address EHR burden. Given these evidence gaps, we seek to contribute to the emerging body of evidence and support the collective effort of reducing EHR burden for all disciplines across countries.

In this viewpoint paper, we describe our efforts to reduce EHR burden for physicians at a large academic mental health hospital in Canada. Building on our previously published studies focused on needs assessment, implementation, and evaluation of individual initiatives [4,14,15], this viewpoint shares the development and implementation of our overarching strategy. We discuss our Physician Engagement Strategy, which aims at identifying and addressing opportunities for EHR improvements at our site. On the basis of our experience to date, we conclude with key success factors and lessons learned in developing and implementing the initiatives included in this strategy.

### The Organization

The Physician Engagement Strategy was implemented in a large academic mental health hospital that provides care to >34,000 patients experiencing mental illness in Toronto, Ontario, Canada. In an effort to improve the quality and continuity of patient care, the organization implemented an integrated EHR system (Cerner Millennium) in 2014. Now, the organization offers a paperless care environment, where all processes, from orders to medication administration, are conducted through and with the EHR system. With a *single source of truth*, the organization has since been able to improve key quality of care outcomes by enhancing medication safety [16] and embedding psychiatric risk flags in the system [17]. This has resulted in obtaining the highest rating (stage 7) on the Electronic Medical Record Adoption Model [18] and level 6 on the Adoption Model for Analytics Maturity [19] from the Healthcare Information and Management Systems Society. Most notably, the organization was awarded the prestigious Davies Enterprise Award in 2018 [20]. On average, the EHR system is used by >400 physicians to deliver care at the organization.

Despite these achievements and the organization’s focus on improving the safety and quality of patient care, physicians have had mounting concerns regarding the usability of the EHR in their daily workflows, highlighting it as a major source of burnout in our 2017 physician wellness survey (conducted with all physicians across the hospital) [15]. Our organization’s wider Physician Engagement, Wellness, and Excellence Strategy included several interventions to improve physician support at the individual, team, and organizational levels. One of the six proposed initiatives under this strategy included *efforts to optimize use of EHRs to enhance the efficiency of practice* [15]. In alignment with departmental leadership (VS) needs to optimize the use of EHR and reduce the associated burden for clinicians, leadership (DJ) from information management prioritized clinician-driven innovation, including efforts to address EHR-related clinician burnout. Consequently, in 2018, the inaugural Chief Medical Information Officer (CMIO) was tasked with improving physicians’ experiences with the EHR to reduce EHR-related burden. Given the importance of leadership buy-in and prioritization of strategies, the CMIO developed and implemented a multipronged strategy to address the ongoing and emerging challenges for physicians related to the use of the EHR.

## Building Our Physician Engagement Strategy

As part of the formative work toward developing and implementing a tailored Physician Engagement Strategy, we undertook a needs assessment in 2019 to understand the main challenges experienced by and EHR-related goals of each academic division within our organization and reviewed the literature to identify strategies and initiatives that could help to address these challenges and aspirations. This effort and the resulting strategy are further described below.

### Needs Assessment

#### Overview

As part of preparatory work to inform and align initiatives targeting EHR burden on physicians to the needs of the organization, it was essential to achieve a holistic understanding of the frustrations and challenges that hinder the effective and meaningful use of the EHR across our organization. A tour of the academic divisions was undertaken and a benchmark survey was launched in 2019 to collect direct feedback and insights on burnout and the current state of EHR use by physicians at our organization. The divisional tour focused on obtaining feedback mostly from physician leadership across the organization, whereas the benchmark survey more broadly and anonymously captured the voice of frontline physicians. This ensured that a balanced *bottom-up* leadership approach was embedded in the development of the strategy. It should be noted that this divisional tour was conducted when several foundational articles (eg, *Why doctors hate their computer* by Gawande [21]) appeared in the published literature highlighting the issues for the medical community, including within the Canadian context [22]. As such, there was great interest from both the organization and frontline physicians to participate in these activities.

#### Divisional Tour

Through the divisional tour, our hospital’s CMIO visited each of the 7 academic physician divisions’ monthly meetings and gathered feedback on their top 3 priorities for EHR optimization. There were 2 main purposes for the divisional tour. First, given that the benchmark survey focused on collecting individual feedback from frontline physicians, the divisional tour helped to contextualize the results through an in-depth discussion with the team. Second, it was also used to obtain buy-in from frontline and clinician leaders. Buy-in was a critical part of the success of these initiatives, and this opportunity provided another forum for physicians to contribute their ideas and perspectives.

In addition to discussions with clinical leadership during these meetings, frontline physicians were also consulted through the existing team huddles for each academic division to obtain their perspective on these issues. It should be noted that this divisional tour was conducted 5 years after the introduction of the hospital’s EHR system. From these consultations, the identified improvement priorities included documentation, orders, and chart navigation through the EHR. More specifically, within *documentation,* there were requests to improve standardized templates and auto-population, increase access to speech recognition technology, and reduce clicks. Second, regarding *orders,* physicians identified the need to increase practice-specific order sets, reduce clicks within workflows, and simplify order measures. Third, in *chart navigation,* there were requests to make the EHR more user-friendly and intuitive with respect to finding relevant information. The automatic inclusion of all laboratory test results and previous medications through the EHR has led to an increase in note sizes that contain multiple pages of nonessential information, which is a phenomenon termed as *note bloat* [23,24], and is frequently identified as a concern for patient safety. Other priorities were also identified, such as the desire to integrate physician billings within the EHR and to simplify the process of discharge medication reconciliation.

#### Benchmark Survey

Following this tour, we conducted a benchmark survey to identify the extent of burnout among our physicians and identify the significant EHR-related contributors to physician burnout [4]. The methods and approach are detailed in a separate paper [4]. We achieved a high survey response rate among full-time physicians (156/208, 75%), with 25.6% (45/176) of physicians reporting having one or more symptoms of burnout. Furthermore, 74.5% (155/208) of respondents who reported burnout symptoms identified the EHR as a contributor. Safety concerns with the EHR and efficient communication were 2 factors that differentiated the groups that were burned out from those that were not. Respondents who had high satisfaction with the EHR used work-arounds to complete tasks (eg, copy and paste from word processing software) or were super users with knowledge of documentation *shortcuts*. Those with low EHR-satisfaction reported excessive clicks and time sinks with using the EHR [4].

Overall, our survey demonstrated that there was a critical need to mitigate EHR-related burden and the associated burnout by optimizing the EHR to fit within physicians’ workflows and by improving awareness of EHR best practices.

### Literature Review

Consistent with academic practices, we sought to inform our Physician Engagement Strategy with the latest evidence. Given the number of interventions aimed at combating EHR-related burnout in the published literature, our team conducted a rapid literature review on this topic [25]. From a review of 50 related articles published between 2014 and 2019, we found that the measurement of EHR burden needs to be performed both subjectively (eg, via surveys) and objectively (eg, using use data) [26]. We also identified that interventions to reduce EHR burnout were focused on four main aspects: enhancing and redesigning the interface of EHR screens, delivering tailored education and training to end users, improving communication, and providing additional support for administrative tasks [25]. To implement many of these interventions, approaches similar to a Plan-Do-Study-Act cycle for quality improvement [27] were used, which was in alignment with our needs assessment process (ie, measuring *pain points*). Through our literature review, we also learned that following the implementation of interventions, it is essential to continue enhancing and optimizing these through further evaluation using analytics data, surveys, and other methods. Although findings from our literature review have laid the foundational building blocks of our strategy, it was important to adapt and align these strategies and interventions to the specific challenges present within our organization.

## The Physician Engagement Strategy

### Overview

**Figure 1:**
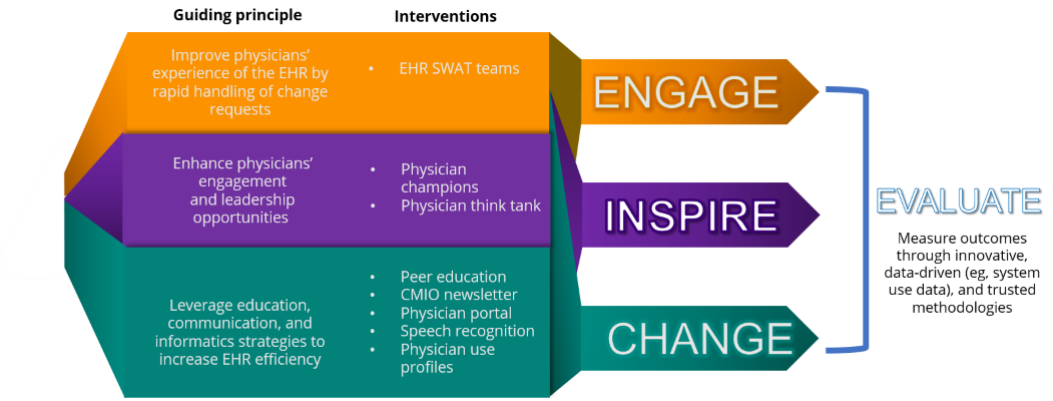
Physician Engagement Strategy. CMIO: Chief Medical Information Officer; EHR: electronic health record.

On the basis of our in-depth needs assessment and review of the literature, we developed our first iteration of the Physician Engagement Strategy in 2020. In the development of this physician-centric multipronged approach, we had in-depth discussions with our information technology (IT) and clinical leadership teams to ensure feasibility and availability of resources. An overview of the strategy is depicted in Figure 1. The strategy comprises four main goals: (1) improving the handling and resolution of EHR issues; (2) enhancing physician engagement and leadership opportunities on EHR-related decisions; (3) leveraging communication, education, and informatics strategies to increase efficient and meaningful use of the EHR; and (4) measuring the impact of these strategies to achieve data-driven insights.

Throughout the implementation of initiatives to support these goals, 3 main guiding principles (pillars) were considered essential for success. Foremost, it was critical to *engage* all relevant stakeholders and frontline physicians to ensure that their perspectives are heard and any challenges are brought up for examination. We also considered it important to provide physicians an opportunity to be *inspired* to participate in leadership roles and be involved in the decision-making process of EHR changes that impact patient care and physicians’ use of the EHR. Finally, we detailed a multitude of initiatives aimed at collectively supporting the ability to *change* the use of the EHR system such that it improves efficiency and end user experience. Throughout these pillars, we also ensured that there was a focus on measuring outcomes to *evaluate* whether the specific initiatives have worked as intended. The initiatives outlined in the strategy are described in detail below.

### Engage: Improve Physician’s Experience With the EHR by Rapid Handling of Change Requests (SWAT Teams)

To engage physicians across our organization, we developed an EHR *SWAT* team initiative through adaptation of initiatives [28] identified from our literature review. Traditional governance models have focused on identifying and implementing requirements solely by the IT team [3]. However, from the IT perspective, there is often a lack of understanding of the actual requirements needed for the change to be impactful. Moreover, many of the changes often affect other clinical areas (eg, pharmacy and laboratory). Thus, implementing in isolation can lead to more downstream challenges. As such, the SWAT team model, which mirrors other initiatives at the University of Colorado School of Medicine [28,29], overcomes this challenge by bringing together a collaborative team to discuss and identify a commonly agreed set of requirements for each issue. Our team-based intervention (*SWAT*) included assembling an interdisciplinary team of specialists including our CMIO; clinical informatics nurses and educators; and representatives from pharmacy informatics, health information management, clinical applications, and project management [14]. Through this intervention, we met with physicians from each of the seven academic divisions across our organization, collected EHR change requests, and prioritized them into four categories: (1) additional education, (2) quick fixes (<6 weeks), (3) future fixes (≥1 year), and (4) unable to address owing to technical or regulatory restraints. In total, we gathered 118 requests (eg, including adding keyword search functionality, minimizing freezing, and auto-faxing) [14].

### Inspire: Enhance Physician’s Engagement and Leadership Opportunities

#### Physician Champions

As part of our focus on inspiring and fostering physicians’ voices in the decision-making process, we designated physician EHR champions (*liaisons*) tasked with liaising with all physicians in their division and bringing forward pain points and recommended changes on an ongoing basis. Divisional liaisons were nominated annually by both their peers and divisional leadership and became the link between different stakeholders, helping us make meaningful EHR changes. These individuals were key players within our EHR SWAT initiative. Liaisons’ responsibilities continued to evolve with the changing needs of the initiatives, and they became the pilot user group for future technology and informatics applications.

#### Physician Think Tanks

Discussions with physician champions highlighted the need for a cross-divisional lens to identify the applicability of EHR changes. As such, a venue to address this gap and facilitate discussion between physician divisional liaisons and other relevant stakeholders was needed. We implemented monthly *Physician Think Tanks* in 2019, which focused on the successful use of the EHR to improve patient safety and quality of care. These meetings are chaired by our CMIO and are attended by physician champions from each academic division and relevant clinical (eg, pharmacy, laboratory, and diagnostics informatics and professional practice) and IT leaders (eg, clinical applications), with composition similar to that of our SWAT team. Before each of these meetings, stakeholders are encouraged to bring forward challenges and questions for discussion at these meetings.

Appropriate engagement with stakeholders is critical for implementing digital tools in a meaningful manner that aligns with the needs of end users, and therefore, we used this forum before the implementation of any new initiatives. As these meetings evolved since their inception in 2019, the venue became a versatile space for EHR optimization. New ideas and features (eg, optimizing order set and reducing auto-population content) are demonstrated at these meetings to collect initial feedback from clinicians. Updates on initiatives (eg, SWAT) and implementations are also presented at these meetings to help support brainstorming of the feasibility and availability of resources. From a quality improvement perspective, evaluation results are presented to solicit feedback from the group for contextualizing the results and identifying next steps for optimization of the EHR.

### Change: Leverage Communication, Education, and Informatics Initiatives to Increase EHR Efficiency

#### Communication: CMIO Monthly Newsletter

One of the main challenges identified from our benchmark survey was the lack of appropriate communication channels for EHR-related updates (eg, policy and technical changes). Although the organization provided EHR updates to all users on a regular basis, these email updates were incomplete and not tailored to physicians. Given that these EHR changes usually coincide with broader organizational policy changes and mandates, physicians reported a lack of a single source of truth for any EHR-related updates. These issues can cause confusion and inconsistency in EHR use across the organization, leading to concerns regarding patient safety and quality of care.

**Figure 2:**
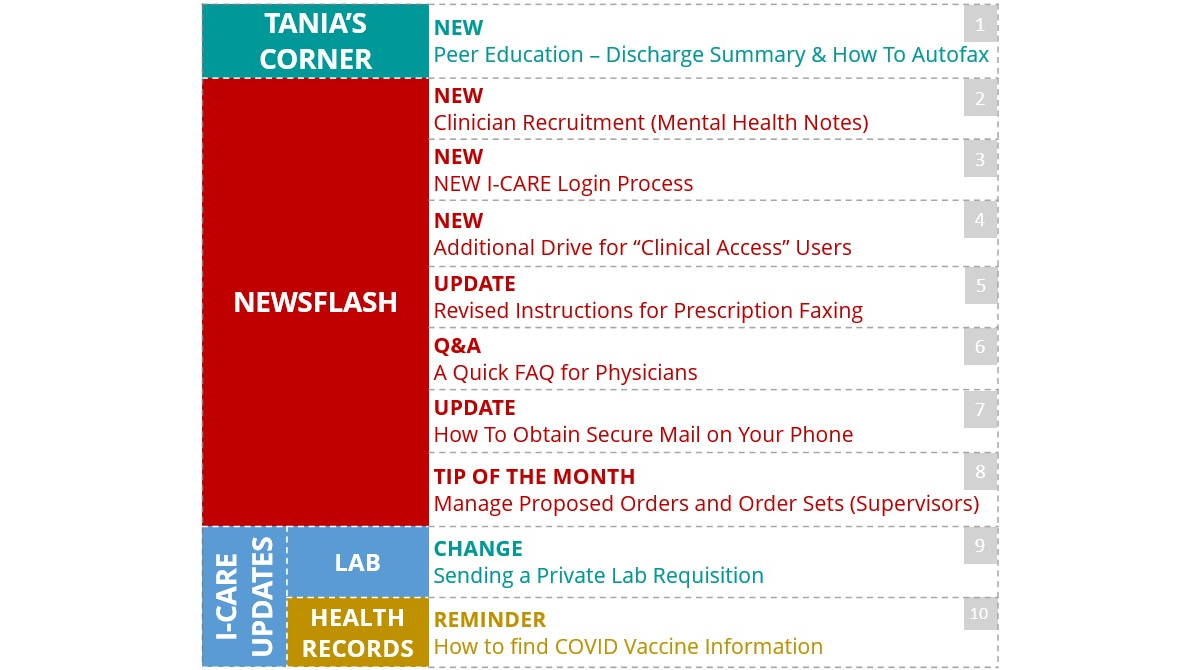
Chief Medical Information Officer monthly newsletter (table of contents). FAQ: frequently asked questions.

To address this issue, the CMIO monthly newsletter was developed. These newsletters are developed with in-depth consultation with clinical (eg, clinical informatics nurse educators) and IT stakeholders to ensure that the content is relevant and useful. The content of the newsletter varies each month and depends on the changes and discussions at that time (Figure 2). Examples of content include initiative updates, interviews with digital health leaders, EHR tips and tricks, related literature, IT changes and EHR changes for health records, pharmacy and therapeutics, and laboratory and diagnostics related EHR updates and clarifications. The newsletter was also a critical method to close the loop of communication on all changes requested through our SWAT initiative. The monthly read rates of these newsletters continue to increase, with our last newsletter (ie, April 2021) being opened by >208 physicians (approximately 50% open rate).

#### Communication: Physician Portal

In addition to the issues related to communication, another intervention was developed to support navigation and rapid finding of EHR-related information. The *Physician Portal* is a collaborative initiative with the physician-in-chief to provide physicians with a one-stop web-based location for all information relevant to physicians, such as wellness initiative updates, policies, and educational presentations. Within the portal’s EHR tips and tricks section, all EHR-related information can be found. Previous CMIO monthly newsletters are made available through this portal, and physicians are able to use a search functionality to find relevant information across the site. Our peer education videos are also hosted on this portal. Recently, tip sheets for EHR use have been added to the site, and physicians are encouraged to visit the site to access these resources. The centralized location for EHR-related information is expected to reduce the navigation burden for EHR-related information.

#### Education: Peer Education Videos

Electronic learning modules have become a staple for providing training and support for a wide range of practice- and policy-related topics. However, their uptake and effectiveness for EHR training and best practices for physicians remain very limited. Given that this user group is inundated with information daily, it was essential to develop targeted messaging that aligns with their needs and questions [30]. As such, peer education was identified to be a useful approach for addressing these issues. Peer education through super users can support end users in mastering the use of the EHR, and previous peer-led EHR training initiatives in Southern California Permanente Medical Group have found that it can save physicians 4 to 5 minutes per hour, equating to approximately 40 minutes a day [31].

Although peer EHR education is typically done in real time and in person, social distancing restrictions of the COVID-19 pandemic have made this difficult to achieve. As a result, we piloted the development of peer education videos. These peer education videos are short in length (3-5 minutes) and focus on specific knowledge gaps found across the organization. During these videos, super users are invited to provide education and guidance (eg, demonstration of a workflow) to the audience based on their experiences and best practices. These videos are posted on our *Physician Portal*, and communication to increase awareness of these tools are done through the monthly CMIO newsletter and the weekly departmental physician newsletter. Physicians are invited to watch the videos in their own time and pace. Currently, two modules have been developed (medication reconciliation and discharge summaries) and a few other videos are in development.

#### Informatics: Speech Recognition Technology

Documentation burden was identified as an important issue for physicians in both the benchmark survey and the divisional tour. In particular, given that psychiatric documentation is fairly narrative in nature, physicians spend significant amount of time typing directly into their EHR system. As a result, documentation methods remain a significant pain point for the organization.

Speech recognition technology has been identified as a suitable solution for mitigating documentation burden in the EHR. Evaluations conducted in other settings have found that physicians report satisfaction with speech recognition technology and its utility in reducing the burden. At our organization, an older version of speech recognition software was procured and deployed to a small number of physicians [32]. However, the limited licenses and lack of a concerted support strategy led to its suboptimal adoption across the organization. Given the renewed focus on this issue, we endeavored to roll out an improved version of speech recognition technology that features improved accuracy, a mobile app microphone, and cloud-based dictation engine. As part of this rollout, all physicians and residents will have access to speech recognition technology for documentation in the EHR system [30].

The speech recognition service will be delivered collaboratively with the other initiatives of the strategy in several ways. The EHR SWAT team intervention will be used to collect technical issues and feature improvements with regard to the platform. The Physician Portal and CMIO newsletters will be used to communicate improvements to the platform and encourage physicians to receive optimization training and education using the platform.

#### Informatics: Physician EHR Use Profiles

**Figure 3:**
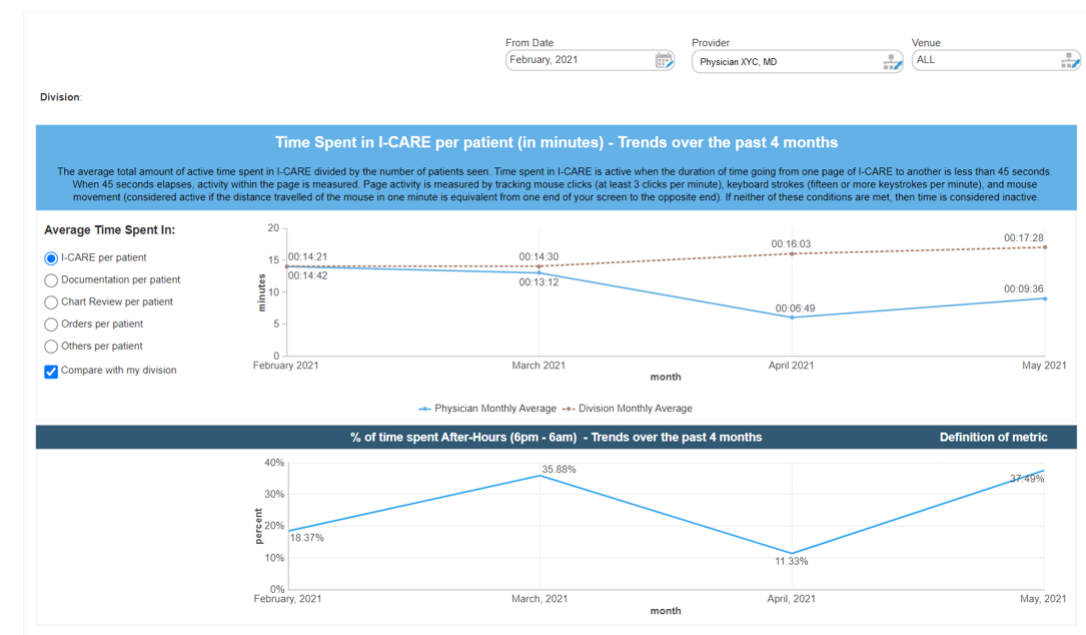
Physician electronic health record use profiles.

Another complementary intervention to improve physicians’ awareness of their EHR practice and deliver feedback was the use of dashboards. Direct feedback dashboards have been used widely across medicine for outcomes such as improving compliance for venous thromboembolism prophylaxis [33], reducing imaging use [34], and improving pediatric emergency care [35]. Dashboards convey information through the use of visual representation of data to help amplify cognition and capitalize on human perceptual capabilities [36]. Our *physician use profiles* (Figure 3) are dashboards that display information on an individual physician level, allowing physicians to view their own EHR system use including metrics such as time spent within the EHR per patient, time spent in documentation, percentage of after-hours use, number of clicks per order, and other measures of efficiency. Through these dashboards, physicians can also compare their EHR use with their divisional average and identify whether they need to lean into the various initiatives of the Physician Engagement Strategy, such as additional training through EHR SWAT meetings, peer education videos, or speech recognition technology. We also anticipate physicians to self-identify as super users through the use of these dashboards (as only the individual physician can look at their own data) and contribute to peer education within their division. Physicians can view their metrics for a specific period (eg, before and after using speech recognition), with provided context as to how each of the metrics is being calculated. Our team is currently in the process of finalizing the design and content of these *physician EHR use profiles* through extensive consultation with the physician divisional liaisons, academic chiefs, and Physician-In-Chief to make it a meaningful and useful intervention.

### Evaluate: Measure Outcomes Through Innovative and Trusted Methodologies

#### Overview

Through our many complementary and interconnected initiatives, we have ensured that time, effort, and resources were used to evaluate each of them. Throughout the development of each intervention, we embedded an evaluation approach to determine whether we have achieved the objectives of the intervention and to identify approaches to streamline or optimize the intervention. This aligns closely with best practices [37] and the Plan-Do-Study-Act cycle [38] of quality improvement. The sources and examples of evaluation initiatives are elaborated in greater detail below.

#### System Use Data

The role of back-end use data has become an important source of information for identifying user-specific challenges within the EHR system and for guiding tailored and customized training. Within our organization, the use of *EHR use metrics* allow us to objectively measure the impact of our initiatives. Our EHR vendor’s back-end analytics platforms (ie, Cerner *Lights On* and Cerner Advance [39]) provide detailed analyses of EHR use for all users including physicians, nurses, and those in other disciplines. Leveraging key metrics such as total time spent per patient in documentation, chart review, ordering, and medication reconciliation of the electronic medical record has allowed us to identify whether a specific intervention (eg, speech recognition technology) has impacted the targeted metric (ie, documentation time). There are several other metrics that can be explored, such as contextual metrics (eg, number of patients that a physician has seen over the past month and the percentage of time physicians spend in the EHR after hours) and workflow metrics (eg, number of clicks used per order and use of order set). Following a thorough validation of how the data within this system compares with our physicians’ actual use, we are slowly beginning to leverage this tool to help learn about our physicians’ experience with the EHR, eventually working to reduce EHR burden.

Similarly, we are also leveraging the use of *system use metrics* from other technologies that we have implemented, such as our speech recognition solution. For example, personalized messages are periodically delivered to physicians who have access to speech recognition technology and are not using the platform, to identify if they require any technical support or additional training.

#### Surveys, Interviews, and Focus Groups

When system use data collection is not feasible or fit for evaluation, we conducted evaluations through surveys, interviews, and focus groups, leveraging specific channels of our strategy such as the physician divisional liaisons where possible. For our EHR SWAT initiative, we used short anonymous surveys (with 5-point Likert scales and free-text questions) following the divisional meeting to gather physicians’ satisfaction with the initiative [14]. In all, 61% (28/46) of the physicians reported that the intervention increased their proficiency in using EHR functionalities [14]. Surveys and interviews were also leveraged to measure the implementation feasibility of speech recognition, complementary to documentation time outcomes measured from system use data.

## Discussion

### Overview

To our knowledge, the Physician Engagement Strategy is one-of-a-kind collaborative approach in a Canadian mental health setting that aims to engage both frontline physicians, physician leadership, and IT leaders in reducing EHR burden. Previous national strategies in the United States to reduce clinician burnout have included recommendations such as engaging clinicians in the design and deployment of health IT to ensure the effectiveness, efficiency, usability, and safety of the technology [9], which we have implemented thoroughly throughout our strategy, especially within the *engage* and *inspire* pillars. The Office of the National Coordinator for Health Information Technology in the United States has also released its Strategy on Reducing Regulatory and Administrative Burden Relating to the Use of Health IT and EHRs, which includes recommendations such as better alignment of the EHR system design with real-world clinical workflows, increase in end user training, and improvement of the clinical documentation functionality [40], which we implemented through our EHR SWAT teams, peer education, and speech recognition initiatives, respectively. Our development of a shared vision and approach enables a concerted strategy developed and implemented with feedback and alignment across all stakeholder groups. As the digital health ecosystem (eg, web-based care) continues to become integral to clinical care, the numerous venues for discussion across multiple departments allow for unbiased feedback and opportunities to align the road maps of the EHR and related technologies.

### Lessons Learned

As we enter the third year of implementing the EHR Physician Engagement Strategy, a few *lessons learned* that will guide future approaches to optimize the reach and impact of our strategy were identified. Foremost, it is essential that frontline physicians are recognized as the main stakeholders and decision makers in our strategy. Across all our initiatives, careful deliberation and stepwise iterative approaches were used to embed physicians’ perspectives and feedback in the implementation and to roll out plans. These stepwise approaches ensured that any *red flags* can be considered and mitigated before the roll out of any initiatives. For example, for speech recognition technology, extensive consultation sessions were conducted before the development of the implementation plan. Understanding physicians’ desires and needs helped curate the main components to consider in such an implementation plan. In addition, these discussions should be considered in multidisciplinary forums. Given that the organization has >70 clinics, each specializing in different diagnoses, treatment, or patient populations, it is expected that significant heterogeneity exists in terms of workflows and EHR use patterns. Ensuring that IT and clinical stakeholders from these clinics are engaged in the decision-making process reduces the possibility of unexpected roadblocks or unintended consequences during implementation. In addition, we realized that periodic review of the initiatives can yield synergistic opportunities to better align and reinforce initiatives within the strategy. In particular, interconnected initiatives can inform each other and maximize their success. For example, during the implementation of speech recognition, the Physician Think Tank was used to identify physicians’ needs before the implementation. In addition, the CMIO newsletter was found to be a useful way to communicate updates and success outcomes to all physicians at the organization. Thus, the initiatives of this strategy enable a concerted effort to reduce EHR burden.

### Future Directions

As we continue to implement and expand the Physician Engagement Strategy, it has become increasingly important to consider this work in the broader field of EHR burden and digital technologies. We expect that this road map will continue to expand as new features and technologies are becoming embedded within the organization. In this accord, we highlight a few important next steps that we hope to achieve in the coming years. First, many of the interventions available in the literature have not focused on evaluating their impact on EHR burden. With the advent of EHR back-end use data, there is a timely opportunity to evaluate the impact of these initiatives on efficiency and satisfaction with using the EHR. In the next year, we will focus on evaluating and determining the impact of these initiatives using our established evaluation methodologies. We will also explore the perceptions and experiences of clinicians in participating in these initiatives (eg, multidisciplinary groups and super users) to address EHR-related burden [37]. In addition, a number of emerging trends, such as measurement-based care [41,42] and web-based care [43], are becoming evident in our digital mental health infrastructure. As these models of care are streamlined, it would be useful to evaluate their impact on EHR burden. Future work should explore how these initiatives can be made useful prospectively in the planning and implementation of these digital models of care at the organizational level. Finally, we are currently exploring opportunities to embed these initiatives as part of creative professional activities for academic promotions. Doing so may help to support the development of *hybrid physicians* [44] who are equipped to enable the effective use of informatics by physicians to deliver better mental health care.

### Conclusions

As EHR-related burden continues to be a critical challenge for many health care systems, we introduce our EHR Physician Engagement Strategy as an approach to reducing these unintended consequences. This strategy involves a multipronged approach that aims to engage clinicians in reimagining their future EHR experiences and empower them as central stakeholders and advocates for digital technologies to achieve the quadruple aim of health care. Future work should focus on evaluating the impact of these initiatives on EHR burden and expanding the impact of this work to other digital health tools at the organization.

## Abbreviations

CMIO: Chief Medical Information Officer
EHR: electronic health record
IT: information technology
